# PROCalcitonin-based algorithm for antibiotic use in Acute Pancreatitis (PROCAP): study protocol for a randomised controlled trial

**DOI:** 10.1186/s13063-019-3549-3

**Published:** 2019-07-29

**Authors:** Ajith K. Siriwardena, Santhalingam Jegatheeswaran, James M. Mason, Minas Baltatzis, Anthony Chan, Aali J. Sheen, Derek O’Reilly, Saurabh Jamdar, Rahul Deshpande, Nicola de Liguori Carino, Thomas Satyadas, Ahmed Qamruddin, Katharine Hayden, Michael J. Parker, John Butler, Azita Rajai, Ben McIntyre

**Affiliations:** 1 Regional Hepato-Pancreato-Biliary Unit, Manchester Royal Infirmary, Oxford Road, Manchester, M13 9WL UK; 2 Faculty of Biology, Health and Life Sciences, University of Manchester, Manchester Academic Health Sciences Centre, Manchester, UK; 3 Warwick Medical School, University of Warwick, Coventry, UK; 4 Centre for Biomedicine, Manchester Metropolitan University, Manchester, UK; 5 Department of Microbiology, Manchester Royal Infirmary, Oxford Road, Manchester, M13 9WL UK; 6 Department of Clinical Biochemistry, Manchester Royal Infirmary, Oxford Road, Manchester, M13 9WL UK; 7 Critical Care Unit, Manchester Royal Infirmary, Oxford Road, Manchester, M13 9WL UK; 8 Pharmacy Department, Manchester Royal Infirmary, Oxford Road, Manchester, M13 9WL UK

**Keywords:** Acute pancreatitis, Antibiotics, Procalcitonin

## Abstract

**Background:**

Differentiating infection from inflammation in acute pancreatitis is difficult, leading to overuse of antibiotics. Procalcitonin (PCT) measurement is a means of distinguishing infection from inflammation as levels rise rapidly in response to a pro-inflammatory stimulus of bacterial origin and normally fall after successful treatment. Algorithms based on PCT measurement can differentiate bacterial sepsis from a systemic inflammatory response. The PROCalcitonin-based algorithm for antibiotic use in Acute Pancreatitis (PROCAP) trial tests the hypothesis that a PCT-based algorithm to guide initiation, continuation and discontinuation of antibiotics will lead to reduced antibiotic use in patients with acute pancreatitis and without an adverse effect on outcome.

**Methods:**

This is a single-centre, randomised, controlled, single-blind, two-arm pragmatic clinical and cost-effectiveness trial. Patients with a clinical diagnosis of acute pancreatitis will be allocated on a 1:1 basis to intervention or standard care. Intervention will involve the use of a PCT-based algorithm to guide antibiotic use. The primary outcome measure will be the binary outcome of antibiotic use during index admission. Secondary outcome measures include: safety non-inferiority endpoint all-cause mortality; days of antibiotic use; clinical infections; new isolates of multiresistant bacteria; duration of inpatient stay; episode-related mortality and cause; quality of life (EuroQol EQ-5D); and cost analysis. A 20% absolute change in antibiotic use would be a clinically important difference. A study with 80% power and 5% significance (two-sided) would require 97 patients in each arm (194 patients in total): the study will aim to recruit 200 patients. Analysis will follow intention-to-treat principles.

**Discussion:**

When complete, PROCAP will be the largest randomised trial of the use of a PCT algorithm to guide initiation, continuation and cessation of antibiotics in acute pancreatitis. PROCAP is the only randomised trial to date to compare standard care of acute pancreatitis as defined by the International Association of Pancreatology/American Pancreatic Association guidelines to patients having standard care but with all antibiotic prescribing decisions based on PCT measurement.

**Trial registration:**

International Standard Randomised Controlled Trial Number, ISRCTN50584992. Registered on 7 February 2018.

**Electronic supplementary material:**

The online version of this article (10.1186/s13063-019-3549-3) contains supplementary material, which is available to authorized users.

## Background

Overuse of antibiotics and the resultant emergence of multidrug-resistant organisms is a potent threat to the welfare of humanity in the twenty-first century [1, 2]. Acute pancreatitis is an inflammatory disorder of the pancreas with an incidence of 150–420 cases per million [3] and an overall case-fatality rate of 4–6% [4, 5]. In addition to being a significant cause of death, severe acute pancreatitis (SAP) is associated with prolonged critical care occupancy, lengthy in-patient stay and slow rehabilitation [6]. SAP is characterised by necrosis of pancreatic tissue which with bacterial colonisation leads to infected necrosis [7]. Antimicrobial therapy to prevent infection of necrosis in acute pancreatitis has been evaluated in a series of randomised controlled trials, with overall findings demonstrating a lack of benefit reported in meta-analyses and a Cochrane systematic review [8–10].

Correct use of antibiotics is important in patients with infected necrosis but use in those with systemic inflammation in acute pancreatitis is non-therapeutic and possibly harmful [11]. Discriminating between pancreatic infection and inflammation is difficult, with neither clinical assessment nor markers of inflammation (such as leukocyte count or C-reactive protein) being sufficiently accurate [12]. As a result there is overuse of antibiotics for suspected infection in acute pancreatitis, with up to two-thirds of patients receiving at least one course of antibiotics during their admission [13]. Antibiotic overuse in acute pancreatitis is widespread not only in the United Kingdom’s National Health Service (NHS) [14] but also worldwide [15–18]. Overuse of antibiotics in acute pancreatitis is associated with the emergence of resistant organisms, antibiotic-related side effects, compromised treatment efficacy and unnecessary health care costs. Nationally, the National Confidential Enquiry into Patient Outcome and Death (NCEPOD) [19] undertook the largest survey to date of the treatment of acute pancreatitis in the NHS. “Treat the Cause”, published in 2016, recommends better support for clinicians making bedside decisions about the use of antibiotics [19]. Specifically, the report highlights the need for assistance in differentiating infection from inflammation and better evidence for initiation, continuation and discontinuation of antibiotics in acute pancreatitis.

One method of distinguishing infection from inflammation is measurement of procalcitonin (PCT) [20]. Historically, calcitonin peptides were thought to be responsible for calcium homeostasis but this is now thought to be a relatively minor physiological role, and a more contemporary appraisal is that procalcitonin is a “hormokine”, sharing characteristics of both hormones and cytokines and having roles in maintaining vascular endothelial tone in response to bacterial infection [20]. The procalcitonin level in the bloodstream of healthy individuals is below the limit of detection (10 pg/ml) using clinical assays. Procalcitonin levels rise rapidly in response to a pro-inflammatory stimulus of bacterial origin and normally fall after successful treatment [21]. PCT is more sensitive than clinical assessment and routine laboratory markers of sepsis (such as leukocyte count and C-reactive protein) in detecting pancreatic infection [22]. Algorithms based on measurement of procalcitonin have emerged as a means of differentiating bacterial sepsis from a systemic inflammatory response in a wide range of settings [23].

A recent Health Technology Assessment (HTA) report evaluated procalcitonin testing as a guide to antibiotic therapy and concluded that further studies are needed before widespread adoption [24]. PCT has also been evaluated as a biomarker in SAP, mainly for early prediction of severity and for identification of patients with a high risk of infected pancreatic necrosis [25].

Qu et al. [26] reported the results of the only randomised controlled trial of a procalcitonin algorithm in severe acute pancreatitis: a single-centre study of 71 patients from China. They compared a PCT-based algorithm for guidance of antibiotic use to routine care in patients with acute pancreatitis. The duration of antibiotic treatment in the PCT-guided group was significantly shorter (10.9 ± 2.8 vs 16.1 ± 2.5 days, *p* < 0.001) without any adverse effects on outcome. Duration of intensive care treatment, overall hospital stay and cost of care were significantly reduced in the PCT-guided group. However, all patients in the control arm were given antibiotics for up to 14 days, which does not reflect current international guideline recommendations. Thus, the findings of this study need to be reproduced in a setting where antibiotic use follows contemporary practice before procalcitonin-based algorithms can be recommended to guide antibiotic use in acute pancreatitis.

The PROCalcitonin-based algorithm for antibiotic use in Acute Pancreatitis (PROCAP) trial tests the hypothesis that a procalcitonin-based algorithm to guide initiation, continuation and discontinuation of antibiotics will lead to reduced antibiotic use in patients with acute pancreatitis without an adverse effect upon outcome.

## Methods and analysis

### Design

This is a single-centre, randomised, controlled, single-blind, two-arm phase III pragmatic clinical and cost-effectiveness trial. Patients will be allocated on a 1:1 basis to intervention and control. Patients, but not clinicians, will be blind to their allocation.

### Participants

Participants will be patients with a clinical diagnosis of acute pancreatitis admitted to the hepato-pancreato-biliary (HPB) service of the Manchester Royal Infirmary (MRI). The MRI is the regional specialist HPB service for the Greater Manchester and Cheshire region, a conurbation of 3.2 million people. Patients admitted directly to the service and those arriving as tertiary transfers from other hospitals will be included as separate strata within the trial design.

### Inclusion criteria

Adult patients presenting with acute pancreatitis admitted or referred to the service will be involved. All acute admissions are screened for potential trial participants. Inclusion criteria include the following:

1. Patients over the age of 18 years of age
2. Valid informed consent
3. A diagnosis of acute pancreatitis requiring two of the following three features [7]:

I. abdominal pain consistent with acute pancreatitis (acute onset of a persistent, severe, epigastric pain often radiating to the back)
II. serum lipase activity (or amylase activity) at least three times greater than the upper limit of normal
III. Characteristic findings of acute pancreatitis on contrast-enhanced computed tomography (CECT), magnetic resonance imaging (MR) or transabdominal ultrasonography

### Exclusion criteria

Exclusion criteria include the following [27, 28]:

1. Patients under the age of 18 years of age

2. Comorbidities requiring prolonged antibiotic therapy—such as infective endocarditis
3. Severely immunocompromised patients—such as those with human immunodeficiency virus and with a CD4 count of less than 200 cells/mm^3^; neutropenic patients (< 500 neutrophils/mm^3^)
4. Patients on immunosuppressive therapy
5. Previous thyroid surgery

### Intervention

**Fig. 1:**
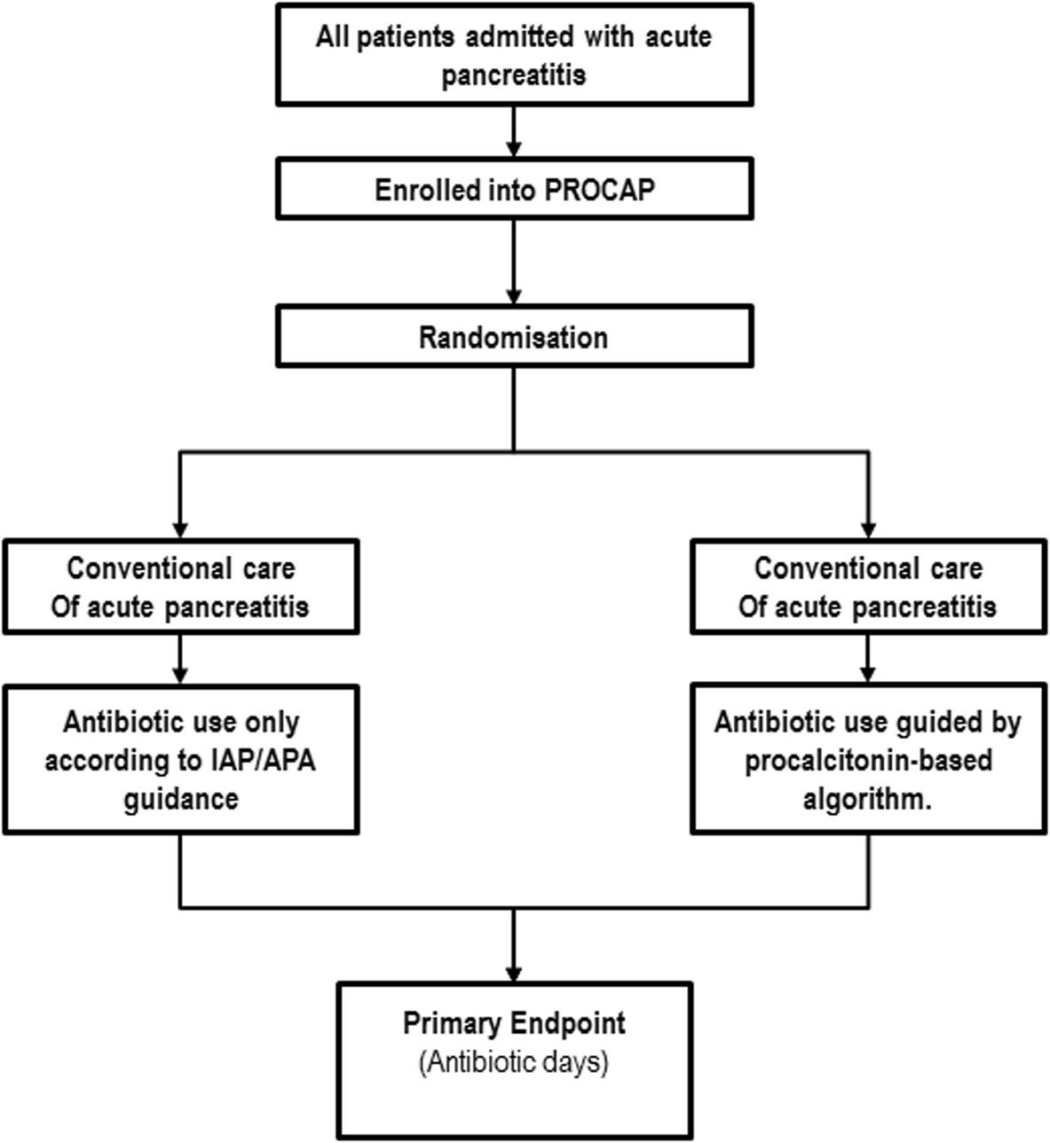
PROCAP study flowchart

The intervention is the use of a procalcitonin-based algorithm to guide antibiotic use. The algorithm is presented in Table 1. A study flowchart is shown in Fig. 1. Patients will be randomised in a 1:1 ratio to receive algorithm-guided or standard care. The randomisation will be stratified by patient admission route (direct or tertiary referral).

**Table 1:** Procalcitonin-based algorithm to guide antimicrobial use in acute pancreatitis

Evaluation only after enrolment at time of admission to hospital		
PCT result	< 1.0 μg/L	≥ 1.0 μg/L
		
Recommendation on antibiotic use	Do not start antibiotics Stop antibiotics in patients already on antimicrobial therapy	Antibiotic intervention (follow Trust guidelines for prophylaxis or treatment)
		
Follow-up	If there is clinical concern about infection, re-check PCT after 24 h	Reassess clinical condition and re-check PCT after 48 h
PCT result	< 1.0 μg/L	≥ 1.0 μg/L
Recommendation on antibiotic use	No antibiotics (or stop antibiotics)	Continue antibiotics (or start if not already on antibiotics) (consider change in antibiotics if clinically indicated)
Over-ruling the algorithm	Empiric antibiotic therapy is permitted in patients not allocated by PCT algorithm to receive antibiotic but decision to be made only by ITU consultant or HPB consultant surgeon and documented in case notes	

*HPB* hepato-pancreato-biliary, *ITU* intensive therapy unit, *PCT* procalcitonin

### Intervention arm protocol

**Fig. 2:**
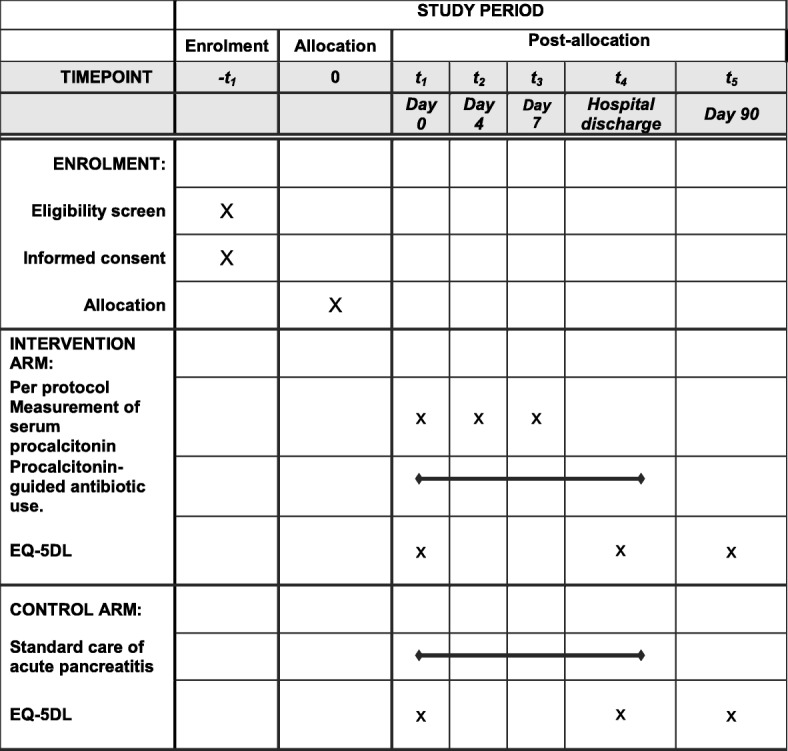
SPIRIT figure for the PROCAP trial. EQ-5DL EuroQol EQ-5D, PROCAP PROCalcitonin-based algorithm for antibiotic use in Acute Pancreatitis, SPIRIT Standard Protocol Items: Recommendations for Interventional Trials

This protocol is summarised in the SPIRIT Figure (Fig. 2). Patients in the intervention arm of the trial are clearly identified by a trial sticker in the case notes and drug kardex. Baseline PCT will be measured on admission (day 0) and the algorithm followed. For patients admitted to ward-based care, PCT will be routinely re-assayed on day 4 and at day 7 after admission for those patients remaining in hospital to these time points. Venesection for PCT assay will be undertaken at the same time as venesection for routine clinical blood tests: no additional venesection is required for PCT measurement. For patients admitted to the critical care unit, PCT will be measured daily during the acute phase of their illness.

Patients who become symptomatic (at any point) for infection will undergo PCT assay and follow the PCT algorithm. Clinically symptomatic patients with a low PCT will not receive antibiotics. If there is persisting concern of infection in patients with a low PCT, the test will be repeated at 24 h. Symptomatic patients with a raised PCT will receive antibiotics according to Manchester University Foundation Trust antibiotic policy. The PCT algorithm will be used to guide continuance and discontinuation of antibiotics. In asymptomatic or symptomatic patients with positive microbiology results, it is appropriate to treat positive microbiology results with antibiotics. PCT should be measured before commencing antibiotics. PCT measurement should be used to guide cessation of therapy, either after 48 h, 72 h or 96 h as clinically appropriate. To avoid repeated short courses of antibiotics, if antibiotic use is triggered by the algorithm, use will be continued for at least 48 h and then PCT re-assayed. If there is no clinical evidence of infection at this point, with this second PCT measurement below threshold, antibiotic use will be discontinued. If antibiotics have been prescribed outwith the algorithm, then continued use will be discussed with the consultant hepato-pancreato-biliary (HPB) surgeon under whose care the patient is being treated or with the Chief Investigator. After this discussion, antibiotic therapy may be stopped. The clinician over-ride can be used to either start or stop antibiotics in situations of clinical urgency. Then, the clinician must be either a consultant HPB surgeon or a consultant intensive care physician, and the reason for over-ride will be documented. If patients undergo endoscopic, radiological or surgical procedures which would normally be undertaken under the cover of antibiotic prophylaxis, it is appropriate to do this without PCT measurement. If prophylaxis is merged with therapy, then the PCT measurement for discontinuance will be triggered.

### Selection of PCT cut-off threshold

As with other biomarkers of severity, the usefulness of PCT is influenced by its chosen threshold value and the timing and accuracy of the assay. Although no absolute consensus exists regarding the most appropriate cut-off value for identification of sepsis in acute pancreatitis, Mofidi et al. [25] report a meta-analysis of eight studies using PCT cut-off values > 0.5 ng/ml. Taken together with the recommendations of Schuetz et al. [23] for PCT algorithms in critical care settings, the optimum threshold for PCT for this study is 1.0 ng/ml.

### PCT assay

The Elecys® BRAHMS fully automated PCT immunoassay (BRAHMS Assay; Roche Diagnostics Ltd, Rotkreutz, Switzerland) will be used for the quantitative determination of procalcitonin in serum.

### Standards of general care for all patients with acute pancreatitis

Standard care will follow the current International Association of Pancreatology/American Pancreatic Association (IAP/APA) guidelines for the care of patients with acute pancreatitis [11]. All aspects of care, with the sole exception of antibiotic use, will be the same for patients in both arms of the trial. As PCT measurement is currently not used regularly in this or other hospitals in the NHS for patients with acute pancreatitis, the control arm will represent current standard care. There will be no procalcitonin measurement in patients allocated to this arm.

### Outcomes

i) ***Primary outcome measure***

The primary outcome measure will be the binary outcome: whether antibiotic use occurs during the index stay.

ii) ***Secondary outcome measures***

1. Safety non-inferiority endpoint all-cause mortality
2. Days of antibiotic use (for antibiotics initiated during the index stay) defined as any day (24-h period) when antibiotics were prescribed on the patient’s drug prescription chart and administered
3. Clinical infections defined according to the Centers for Disease Control [29]
4. New isolates of multiresistant bacteria (*Clostridioides difficile*, vancomycin-resistant enterococcus (VRE), methicillin-resistant *Staphylococcus aureus* (MRSA), carbapenemase-producing enterobacteriaceae (CPE))
5. Incidence of multiresistant organism bacteraemia
6. Infection of pancreatic necrosis—defined either as a result of fine needle aspiration (FNA), radiological evidence of gas in a peri-pancreatic collection or positive microbiological cultures from surgical or post-mortem specimens
7. Use of radiological, endoscopic or surgical intervention [30]
8. Survival at 90 days; time-to-event (mortality) survival (Kaplan–Maier)
9. Length of inpatient stay (in total and by level of care: critical care levels II/III, ward-based care)
10. Re-admission to hospital within 6 weeks of onset of index episode
11. Episode-related mortality and cause
12. Quality of life assessed by the EQ-5D-5L questionnaire, at enrollment, discharge and 90 days [31]
13. Cost analysis (from an NHS perspective, including inpatient resource use)

iii) ***Measurement of outcomes***

The primary (superiority) outcome measure will be antibiotic use (binary endpoint: yes or no) during the index stay. Antibiotics prescribed before the index admission (from the referring hospital or community) will be recorded at admission but not included in the primary endpoint.

### Sample size

Based on current audit data, 60% of patients admitted with acute pancreatitis receive antibiotics [13]. A 20% absolute change in antibiotic use would be a clinically important difference. This effect of intervention has been observed in other studies evaluating a procalcitonin algorithm to guide antibiotic use [23, 28]. A study with 80% power and 5% significance (two-sided) would require 97 patients in each arm (194 patients in total). The study will aim to recruit 200 patients. Assuming a 3.6% mortality rate based on unit audit data [13], the sample size provides a 6.6% non-inferiority margin for the safety measure of overall mortality, assuming no change in mortality, 80% power and 95% CI (one-sided). Previous randomised trials of the use of a procalcitonin measurement algorithm to guide antibiotic use in a range of clinical settings as well as a Health Technology Assessment have shown no evidence of harm from this intervention [23, 24]. However, as there has been no previous evaluation of procalcitonin in an all-comers population of patients with acute pancreatitis, it is useful to include a secondary safety outcome with mortality being the most important factor. The choice of a 6.6% non-inferiority margin is a pragmatic choice based on the projected sample size and the estimated population mortality rate of 3.6%.

### Consent process

Valid consent will be obtained for all patients. Consent procedures will be governed by the Medicines for Human Use (Clinical Trials) Regulations (2004); Schedule 1, part 5 and by The Medicines for Human Use (Clinical Trials) Amendment (No. 2) Regulations 2006, No. 2984 [32, 33]. Where available, Trust-appointed translator services will be used for those patients who are unable to speak or comprehend English (Additional files 1, 2, 3, 4, 5 and 6).

### Informed consent for patients with capacity

For eligible patients who possess mental capacity, a member of the research team will make the initial approach and provide a verbal overview of the study and what participation will involve. The patient will be provided with a written information sheet and given the opportunity to ask questions. After their questions have been answered, they will have sufficient time to consider participation; if they are willing to take part in the study, they will be asked to sign the consent form.

### Consent procedures for patients who lack capacity

As acute pancreatitis may be severe, causing disruption of a patient’s cognitive state or requiring sedation to facilitate advanced organ support in intensive care, some potential participants will lack capacity to consent for enrolment. Such patients may still be enrolled in this study according to the following procedures. Firstly, a treating clinician who is not part of the study team will assess the competence of a potential participant to consent for research. If lack of capacity to consent is confirmed, then valid consent for enrolment may be obtained from a patient’s legal representative. Ideally, this legal representative will be someone who knows the patient and is able to judge whether the patient would have agreed to enrolment in this study. This personal legal representative would usually be their next of kin or someone with whom they had a significant relationship, and is willing to engage with the consent process on the patient’s behalf. If a personal legal representative is not available, then the patient’s professional legal representative may provide consent instead. This will be an independent treating clinician who is not part of the study team. Where a researcher is also the treating health professional, another member of the research team, independent of any responsibility for the clinical care of that patient, will be asked to make the initial approach and/or seek consent from participants or their legal representative. Patients who recover sufficiently to understand the explanation of the study will be asked to consent to continue with the study procedures as soon as possible or be offered the chance to withdraw. If the patient chooses to withdraw from the study procedures, they will be asked for permission to use their study-related data and for permission to collect and use outcome data. For all participants, written consent forms will be signed; their name filled in and personally dated by the patient or by their legal representative and by the Investigator who conducted the consent discussion. A copy of the signed and dated consent form will be provided to the patient and/or their legal representative and another copy filed in the patient’s medical record.

### Withdrawal of consent

Patients can withdraw consent for participation at any time after enrolment. They do not need to give a reason and their clinical care will be unaffected. Patients allocated to the procalcitonin arm will not continue to undergo PCT monitoring of antibiotic use after withdrawal. Data provided up until the time of withdrawal will be retained for use in analyses.

### Randomisation

Web-based randomisation will be provided by the Clinical Trials Unit of the University of Edinburgh (https://www.ed.ac.uk/usher/edinburgh-clinical-trials). Allocation will be in a ratio of 1:1 to routine or algorithm-guided care. Randomisation will be stratified by disease severity (mild or moderately severe/severe) and admission pathway (whether or not the patient has their index (first) admission with acute pancreatitis to the Manchester Royal Infirmary (direct) or is transferred from another hospital (tertiary transfer)). A random block size of 4, 6 or 8 will be applied to each stratum. Patients allocated to either arm will be identified by a label placed inside and on the front of the case notes with copy labels used for ward folders.

### Data collection

Data collection will use a case report form (CRF) and include source-verifiable data from patient records, including procalcitonin test findings and the list of primary and secondary endpoints. CRFs will be anonymised and contain no individual patient-identifiable information. Patient-level data will be stored by screening and trial log numbers. Data will be recorded on the timeline of the episode, including time from onset of symptoms to admission, days in baseline hospital for tertiary transfers and delay from admission to enrolment. Patients who are discharged and re-admitted within 6 weeks will be regarded as a re-admission for the same episode of care and treatment will be summated. Patients who are re-admitted will be in the same arm as their original allocation. Re-admission elsewhere will be a specific question sought at follow-up (typically at 6 weeks and 90 days). Patients discharged but subsequently admitted elsewhere within 6 weeks will have their pharmacy charts reviewed wherever possible and antibiotic use summated. The trial process and data collection are designed to be minimally burdensome to patients. Clinically, the 90-day follow-up period will complete the involvement of the patient in the trial.

### Data storage and transfer

Paper copies of the CRFs will be stored in a locked cabinet in the Chief Investigator’s office within the Manchester University Foundation Trust. These copies will be stored for 12 months after completion of the trial and then destroyed.

Data will be contemporaneously stored in a password-protected database, allowing ongoing monitoring of data quality and completeness. These data will be stored on a secure desktop computer maintained in the office of the Principal Investigator.

Anonymised data transfer for analysis will be sent electronically only to NHS and university email addresses as anonymised password-protected data.

### Analysis plan

Clinical and economic analysis will follow intention-to-treat principles, as detailed prospectively in a Statistical Analysis Plan. Endpoints will be assessed using an appropriate general linear model adjusted for stratification factors; for the primary endpoint, a general linear regression with logit link will be employed. Missing values will be addressed by multiple imputation, having appropriately explored the missingness mechanism, and in accordance with good practice [34]. Chance baseline imbalances and protocol adherence will be explored within sensitivity analyses.

The trial will determine whether the use of a procalcitonin algorithm reduces antibiotic use during acute pancreatitis. Currently, there is clinical uncertainty about guidelines for reducing antibiotic use in this patient group, since this draws on indirect evidence. Hence, a superiority design has been selected, with a null hypothesis that antibiotic use is unchanged by use of the algorithm.

All-cause mortality in 90 days, re-admission within 6 weeks, adverse events (AEs) and serious adverse events (SAEs) will be reported and compared between the two groups. Length of stay in hospital will be reported and compared using a suitable method (according to its distribution). Other secondary outcomes will be reported using appropriate summary statistics.

Data cleaning and analysis will be provided by the study statistician. Analysis will follow intention-to-treat principles, with patients analysed according to randomisation and irrespective of actual use or compliance with the algorithm. Every effort will be made to retain and include all patients who are part of the trial. Data will be analysed using the latest version of STATA (StataCorp).

Economic analysis will be conducted from an NHS perspective, following similar principles and practices to the analysis of clinical outcomes. Analysis of costs will be limited to hospital activity since these will predominantly determine patient costs during the 90-day period. A within-trial economic analysis will use bootstrapped, adjusted, bivariate regression modelling of costs and quality-adjusted life-years (QALYs), adjusted for baseline scores and stratifying variables [35]. Analyses will be presented as an incremental cost-effectiveness plane, as a cost-effectiveness acceptability curve and by net monetary benefit. An expected value of perfect information (EVPI) analysis will also be provided. Given the timeframe of 6 weeks, discounting of future costs and benefits will not be applied.

### Data Monitoring Committee

An independent Data Monitoring Committee (DMC) will work in accordance with a DAMOCLES charter agreed before trial commencement [36]. It is anticipated that the committee will constitute an independent chair, a statistician and a patient advocate. The DMC will consider protocol adherence, trial withdrawal and safety monitoring, and will make recommendations for continuation of the trial.

The DMC will convene prior to commencement of the trial and at 6-monthly intervals. Recommendations for study continuation, modification or termination will be provided in a confidential report to the Trial Steering Committee.

### Trial Steering Committee

The Trial Steering Committee will include an independent chairperson and an independent member together with the Principal Investigator, trial coordinator and statistician. The TSC will meet every 6 months.

### Governance arrangements

The sponsor will put in place monitoring and oversight arrangements appropriate to the needs of the trial.

### Adverse event reporting

An adverse event is defined as “any untoward medical occurrence that may present during the conduct of the trial, not necessarily having a causal relationship with the intervention being investigated” [37, 38]. An adverse event can therefore be any unfavourable and unintended sign, symptom or disease temporally associated with the trial.

All adverse events will be assessed for:

1. seriousness
2. causality
3. expectedness

The research fellow will notify the Principle Investigator of the adverse event. The Principle Investigator will determine whether it is an adverse event or a serious adverse event. All adverse events will be recorded in line with European Directive 2001/20/EC [39] and recorded in the case report form. An annual safety report to the Data Monitoring Committee will be submitted. SAEs will be reported by email to the Trust quarterly.

### Stopping rules

The trial may be stopped temporarily or permanently (following discussion with the DMC and sponsor) at any point if the following occur [40]:

1. if there is evidence of trial misconduct noted by the DMC or by the MFT Research and Innovation Department
2. if there is evidence of futility or if there is evidence that the safety non-inferiority endpoint is not met
3. if external scientific evidence emerges to render the findings of this trial obsolete or irrelevant

### Study timeline

The study opened on the 26 July 2018 and it is intended to recruit for 2 years subject to satisfactory progress assessment by the DMC and the TSC.

## Ethics and dissemination

### Ethics review

The study was approved by the NHS Health Research Authority (REC reference 18/NW/0255) on 29 May 2018. Site-specific approval was granted by the Manchester University Foundation Trust (Pin B00007) on 5 June 2018.

### Study registration

PROCAP was registered with the International Standard Randomised Controlled Trial Number (ISRCTN 50584992) on 7 February 2018 prior to opening the study for recruitment.

### Reporting of results

Results will be reported at appropriate national and international meetings and published in peer-reviewed journals. The authors undertake to report the results of the completed trial.

### Role of the sponsor and funding

There is no external funding for this study. Costs incurred for registration of the study with the ISRCTN, web-based randomisation and the expenses of the Data Monitoring Committee were met from a Pancreatic Research Endowment fund (9033). The study sponsor had no involvement in study design. The sponsor is not involved in collection, management, analysis or interpretation of the data.

The sponsor will not be involved in writing the report of the decision to submit the report for publication.

The ultimate authority and responsibility for these activities rests with the Chief Investigator.

### Trial sponsor

Name and contact information for the trial sponsor:

Lynn Webster, Head of Research Office, Manchester University NHS Foundation Trust, Manchester M13 9RN, UK. Email: lynn.webster@mft.nhs.uk

## Discussion

The PROCAP trial tests the hypothesis that a procalcitonin-based algorithm to guide initiation, continuation and discontinuation of antibiotics will lead to reduced antibiotic use in patients with acute pancreatitis without an adverse effect upon outcome. The trial explores the concept that a potentially clinically relevant study can be undertaken without peer-review funding but can address an important question.

When complete, PROCAP will be the largest randomised trial of the use of a procalcitonin algorithm to guide initiation, continuation and cessation of antibiotics in acute pancreatitis. PROCAP is the only randomised trial to date to compare standard care of acute pancreatitis as defined by the IAP/APA guidelines to patients having standard care but with all antibiotic prescribing decisions based on procalcitonin measurement.

## Trial status

The current version of the PROCAP protocol is version 1.0. This version was submitted to the North West Research Ethics Committee on 14 March 2018.

The PROCAP trial opened to recruitment on 26 July 2018 and will close to recruitment in July 2020.

## Additional files

Additional file 1: Participant information sheet (DOCX 76 kb)

Additional file 2: Patient consent form (DOCX 53 kb)

Additional file 3: Information to GP (DOCX 22 kb)

Additional file 4: Information for consultee (DOCX 42 kb)

Additional file 5: Consultee declaration form (DOC 40 kb)

Additional file 6: SPIRIT 2013 checklist: recommended items to address in a clinical trial protocol and related documents (DOC 160 kb)

## Data Availability

The dataset used during the current study will be made available from the corresponding author on reasonable request.

## Abbreviations

AE: Adverse event
APA: American Pancreatic Association
CD: Cluster of differentiation
CECT: Contrast-enhanced computed tomography
CI: Confidence interval
CPE: Carbapenemase-producing enterobacteriaceae
CRF: Case report form
DMC: Data Monitoring Committee
EQ-5D-5L: EuroQol EQ-5D
FNA: Fine needle aspiration
HPB: Hepato-pancreato-biliary
HTA: Health Technology Assessment
IAP: International Association of Pancreatology
ISRCTN: International Standard Randomised Controlled Trial Number
MFT: Manchester University Foundation Trust
MR: Magnetic resonance
MRI: Manchester Royal Infirmary
MRSA: Methicillin-resistant *Staphylococcus aureus*
NCEPOD: National Confidential Enquiry into Patient Outcome and Death
NHS: National Health Service
PCT: Procalcitonin
QALY: Quality-adjusted life-year
REC: Research Ethics Committee
SAE: Serious adverse event
SAP: Severe acute pancreatitis
SPIRIT: Standard Protocol Items: Recommendations for Interventional Trials
TSC: Trial Steering Committee
VRE: Vancomycin-resistant enterococcus
