# Supplementary material for: PROCalcitonin-based algorithm for antibiotic use in Acute Pancreatitis (PROCAP): study protocol for a randomised controlled trial
Source: Trials. 2019 Jul 29;20:463. doi: 10.1186/s13063-019-3549-3 (PMC6664733; doi:10.1186/s13063-019-3549-3)
Supplement: Supplementary file 1 — Participant information sheet (DOCX 76 kb) [file 13063_2019_3549_MOESM1_ESM.docx]

#
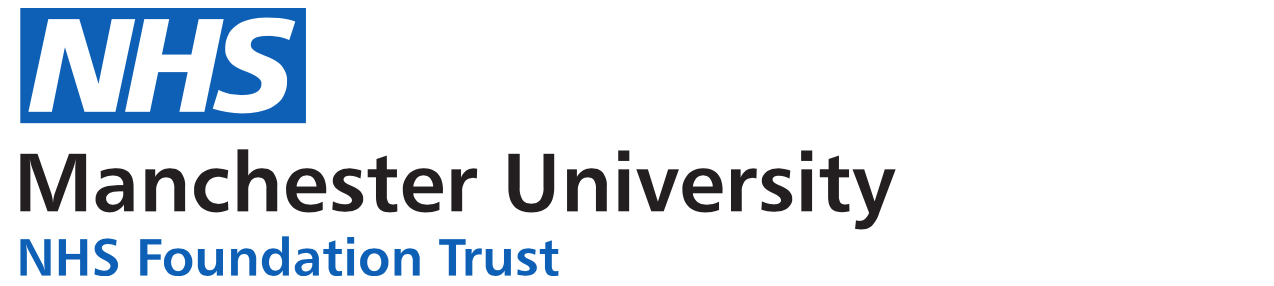


# PARTICIPANT INFORMATION SHEET

**PROCalcitonin-based algorithm for antibiotic use in Acute Pancreatitis (PROCAP): A randomised controlled trial*.***

We would like to invite you to take part in our research study. Before you decide we would like you to understand why the research is being done and what it would involve for you. One of our team will go through the information sheet with you and answer any questions you have. Talk to others about the study if you wish. Part 1 tells you the purpose of this study and what will happen to you if you take part. Part 2 gives you more detailed information about the conduct of the study. Please ask us if there is anything that is not clear.

**Part 1**

The pancreas is an organ in your upper abdomen sitting below the stomach. Its function is to produce enzymes to digest the food that you eat, as well as producing hormones to control your blood sugars. When the pancreas becomes inflamed, it can make you unwell by causing pain and sickness, as well as affecting other vital organs. This condition is termed ‘acute pancreatitis’. The severity of acute pancreatitis can range from being mild to very severe where some patients spend a long time in hospital.

We treat acute pancreatitis mainly by rehydration, as well as giving pain relief and anti-sickness medications. The inflammation usually settles on its own. However, for the small minority of patients who are unfortunate to develop a severe form of acute pancreatitis, bacterial infection around the pancreas can be a problem, and this may require additional treatment such as antibiotics.

***What is the purpose of the study?***

For the doctors looking after you, telling the difference between inflammation and bacterial infection in acute pancreatitis can be difficult. Both conditions can give you a fever and a fast heart rate. Antibiotics are often prescribed where there is no bacterial infection, so there will be no benefit to patients, but can cause harmful side-effects such as severe diarrhea and inflammation of the bowel. There is also a risk of developing bacteria that are resistant to the common antibiotics that we use (so-called ‘*superbugs*’), limiting our choice of antibiotics should you need them in the future.

It is therefore important to only give antibiotics to those patients who have a bacterial infection. There are blood tests, such as Procalcitonin, that can help us distinguish between inflammation and infection. The purpose of our study is to see how effective Procalcitonin is at guiding doctors in using antibiotics in acute pancreatitis.

***What is Procalcitonin?***

Procalcitonin is produced by the cells in our body in response to infection. This can be measured in blood samples. At the moment, it can be used to guide antibiotic treatment in common conditions such as chest infections. Its role for patients with acute pancreatitis who are suspected of having infection, however, is not yet clear.

***Why have I been invited to take part in this study?***

You have been admitted to hospital with acute pancreatitis. There may be a need for antibiotics as part of your treatment if you develop an infection. We would like to measure your Procalcitonin levels to help guide your treatment.

***Do I have to take part?***

Taking part is entirely voluntary, and it is up to you to decide if you want to join the study. We will describe the study and go through this information sheet. If you agree to take part, we will then ask you to sign a consent form. You are free to withdraw at any time, without giving a reason, and this would not affect the standard of care you receive.

***What happens if I wish to take part, and what will I have to do?***

If you agree to take part in the study, we will firstly gather information about the treatment you have already received for acute pancreatitis from your hospital notes.

A ‘randomised controlled trial’ is a study where participants are randomly assigned into one of usually two treatment groups to see which one has a better outcome. In our study, all patients will receive the standard treatment for acute pancreatitis, but will be allocated into one of two groups that differ only in how we decide about antibiotic treatment.

**Group 1:** Treatment with antibiotics will be guided by your Procalcitonin levels, which will be measured from your routine blood tests as part of your treatment.

**Group 2:** Treatment with antibiotics will depend on the clinical judgement of your doctors

For both groups, we will record details of the treatment you receive. The main outcome we are interested in is the number of days you receive antibiotic treatment.

We will also ask you to fill out and return a short questionnaire to measure your quality of life before you are discharged from the hospital.

***What are the possible advantages of taking part?***

We hope to see a safe reduction the use of antibiotics in patients where we measure Procalcitonin levels. We cannot promise the study will help you but the information we get from this study may help improve the treatment of people with acute pancreatitis in the future.

***What are the possible disadvantages and risks of taking part?***

No blood test is perfect, including procalcitonin, and this may still lead to inappropriate antibiotic use. However, if there is a proven infection, then you will receive antibiotics regardless of your Procalcitonin levels if your doctor feels it is necessary.

***What if there is a problem?***

Any complaint about the way you have been dealt with during the study or any possible harm you might suffer will be addressed. The detailed information on this is given in Part 2.

***Will my personal details be kept confidential?***

Yes. We will follow ethical and legal practice and all information about you will be handled in confidence. The details are included in Part 2.

***Who will have access to my personal details?***

Your personal details will be kept anonymous and the research team will only have access to your personal details on a strictly need-to-know basis. All members of the team are bound by patient confidentiality.

**This completes part 1. If the information in Part 1 has interested you and you are considering participation, please read the additional information in Part 2 before making any decision.**

**PART 2**

***What will happen if I don’t want to carry on with the study?***

You can decide at any time to withdraw from the study, even after you have signed the consent forms. Your treatment and the course of your hospital stay will not change in any way if you decide not to take part in the study.

If you withdraw from the study, we will need to use the data (information) collected up to your withdrawal. However, we will not gather further data about your treatment as part of this trial.

If you have been allocated to the group where antibiotic use is guided by Procalcitonin levels, your care will return to normal practice where antibiotic use is guided by the clinical judgement of your doctor.

***What if there is a problem?***

If you have a concern about any aspect of this study, you should ask to speak to either Professor Siriwardena or any member of his research team who will do their best to answer your questions. They can be contacted via Professor Siriwardena’s secretary on 0161 276 4244. If you remain unhappy and wish to complain formally, you can do this via the NHS Complaints Procedure. Details can be obtained from the Patient Advice and Liaison Service (PALS) here at Manchester Royal Infirmary. PALS can also be contacted on 0161 276 8686.

***Will my taking part in this study be kept confidential?***

Yes. Our procedures for handling, processing, storage and destruction of your personal data follow the Caldecott principles (a set of guidelines in the NHS confidentiality code of practice to protect your confidentiality).

All information which is collected about you during the course of the research will be kept strictly confidential, and any information about you which leaves the hospital/surgery will have your name and address removed so that you cannot be recognised. Any data we collect about you that can identify you will be stored securely and will not be disclosed to anyone outside the research group. The only exception to this is the data may be looked at by authorised people to check that the study is being carried out correctly. All will have a duty of confidentiality to you as a research participant and we will do our best to meet this duty.

Any data that can be used to identify you personally will be destroyed at the end of the study. All the data that we use in our analysis will be done so in an anonymised form, and used in a way that will not personally identify you. At the end of the study, we will aim to publish the results of our research in a public domain such as a paper in a medical journal or at a medical conference. No results that we publish will contain information that can personally identify you. The data we collect may also be used to plan further research in this area. Again, this will not include any information that can personally identify you.

***Will my GP be informed if I participate in the study?***

Yes, with your consent, we will write a letter to your GP informing them of your participation.

***What will happen to the information collected about me?***

The information collected about you, the treatment you receive and your quality of life will be collated and analysed during the course of the study. Only the research team who will be working on this project will have direct access to the data.

***What will happen to the results of the research study?***

At the end of the study, we will aim to publish the results in a public domain such as a paper in a medical journal and/or at a medical conference. No results that we publish will contain information that can personally identify you. The results and conclusions from this study may also be used to plan further research.

***Who is organising and funding the research?***

Professor Ajith Siriwardena is organising the research. The research is being funded locally by the Department of Surgery at Manchester Royal Infirmary, Manchester University NHS Foundation Trust.

***Who has reviewed the study?***

All research in the NHS is looked at by independent group of people, called a Research Ethics Committee, to protect your interests. This study has been reviewed and given favourable opinion by the XXX Research Ethics Committee.

***Where can I get more information about the study?***

More general information about medical research can be found at the NHS website (<http://www.nhs.uk/Conditions/Clinical-trials/Pages/Introduction.aspx>). Specific information about this research project, or advice about your participation, can be provided by any member of the research team whom you can ask about any aspect of this study either during your hospital stay, or any at time after your leave hospital.

If you are unhappy about any aspect of this study or have any questions about it, you can contact Professor Siriwardena (via his secretary on 0161 276 4244) to discuss these issues further.
