# Supplementary material for: PROCalcitonin-based algorithm for antibiotic use in Acute Pancreatitis (PROCAP): study protocol for a randomised controlled trial
Source: Trials. 2019 Jul 29;20:463. doi: 10.1186/s13063-019-3549-3 (PMC6664733; doi:10.1186/s13063-019-3549-3)
Supplement: Supplementary file 2 — Patient consent form (DOCX 53 kb) [file 13063_2019_3549_MOESM2_ESM.docx]

#

#
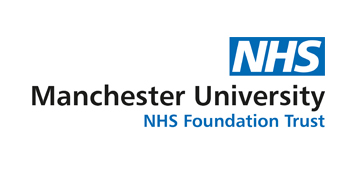


# PATIENT CONSENT FORM.

**Title of Project: PROCalcitonin-based algorithm for antibiotic use in Acute Pancreatitis (PROCAP): A randomised controlled trial.**

**Name of Principal Investigator:**

Professor A K Siriwardena

**Affix Patient Sticker**

**Study Number:**

**Patient ID for this trial:**

**Please initial box**

1. I confirm that I have read and understand the information sheet dated 02/02/2018 (version 1.0) for the above study. I have had the opportunity to consider the information, ask questions and have had these answered satisfactorily.
2. I understand that my participation is voluntary and that I am free to withdraw at any time without giving any reason, without my medical care or legal rights being affected.
3. I understand that relevant sections of my medical notes and data collected during the study may be looked at by individuals from Manchester University NHS Foundation Trust or from regulatory authorities, where it is relevant to my taking part in this research. I give permission for these individuals to have access to my records.
4. I understand the data collected will be kept for up to 1 year.
5. I agree to my GP being informed of my participation in the study.
6. I agree to take part in the above study.

. .

**Patient Signature Print Name Date**

. .

**Consenting Clinician Print Name Date**

When completed: 1 for participant; 1 for researcher site file; 1 (original) to be kept in medical notes.
