# Supplementary material for: PROCalcitonin-based algorithm for antibiotic use in Acute Pancreatitis (PROCAP): study protocol for a randomised controlled trial
Source: Trials. 2019 Jul 29;20:463. doi: 10.1186/s13063-019-3549-3 (PMC6664733; doi:10.1186/s13063-019-3549-3)
Supplement: Supplementary file 4 — Information for consultee (DOCX 42 kb) [file 13063_2019_3549_MOESM4_ESM.docx]

#

#
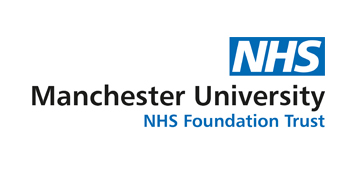


**Study Title: PROCalcitonin-based algorithm for antibiotic use in Acute Pancreatitis (PROCAP): A randomised controlled trial.**

**Information for Consultee**

**Introduction**

We feel your relative/friend is unable to decide for himself/herself whether to participate in this research.

To help decide if he/she should join the study, we’d like to ask your opinion whether or not they would want to be involved. We’d ask you to consider what you know of their wishes and feelings, and to consider their interests. Please let us know of any advance decisions they may have made about participating in research. These should take precedence.

If you decide your relative/friend would have no objection to taking part we will ask you to read and sign the consultee declaration on the last page of this information leaflet. We’ll then give you a copy to keep. We will keep you fully informed during the study so you can let us know if you have any concerns or you think your relative/friend should be withdrawn.

If you decide that your friend/relative would not wish to take part it will not affect the standard of care they receive in any way.

If you are unsure about taking the role of consultee you may seek independent advice.

We will understand if you do not want to take on this responsibility.

The following information is the same as would have been provided to your relative/friend.

*Continue with text from participant information sheet edited where necessary to make sense for the consultee.*
