# Supplementary material for: PROCalcitonin-based algorithm for antibiotic use in Acute Pancreatitis (PROCAP): study protocol for a randomised controlled trial
Source: Trials. 2019 Jul 29;20:463. doi: 10.1186/s13063-019-3549-3 (PMC6664733; doi:10.1186/s13063-019-3549-3)
Supplement: Supplementary file 5 — Consultee declaration form (DOC 40 kb) [file 13063_2019_3549_MOESM5_ESM.doc]

## A possible template for a consultee declaration form for research conducted under the Mental Capacity Act 2005

***(Form to be on headed paper)***

Centre Number: Study Number:

Participant Identification Number for this study:

**CONSULTEE DECLARATION FORM**

**Title of Project:**

**Name of Researcher:**

**Please initial box**

I [name of consultee] have been consulted about [name of potential participant]’s

participation in this research project. I have had the opportunity to ask questions

about the study and understand what is involved.

In my opinion he/she would have no objection to taking part in the above study.

I understand that I can request he/she is withdrawn from the study at any time,

without giving any reason and without his/her care or legal rights being affected.

I understand that relevant sections of his/her care record and data collected during the study

may be looked at by responsible individuals from [name of sponsor and/or host organisation]

or from regulatory authorities, where it is relevant to their taking part in this research.

I agree to their GP or other care professional being informed of their participation in the study.

Name of Consultee Date Signature

Relationship to participant:

Person undertaking consultation (if different from researcher):

Name Date Signature

Researcher Date Signature

When completed: 1 (original) to be kept in care record, 1 for consultee; 1 for researcher site file
