# Supplementary material for: PROCalcitonin-based algorithm for antibiotic use in Acute Pancreatitis (PROCAP): study protocol for a randomised controlled trial
Source: Trials. 2019 Jul 29;20:463. doi: 10.1186/s13063-019-3549-3 (PMC6664733; doi:10.1186/s13063-019-3549-3)
Supplement: Supplementary file 6 — SPIRIT 2013 checklist: recommended items to address in a clinical trial protocol and related documents (DOC 160 kb) [file 13063_2019_3549_MOESM6_ESM.doc]

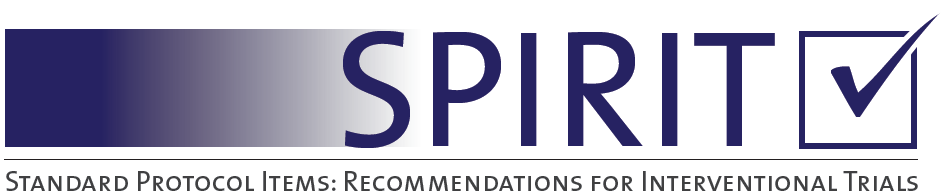


SPIRIT 2013 Checklist: Recommended items to address in a clinical trial protocol and related documents*

| Section/item | ItemNo | Description |
| --- | --- | --- |
| **Administrative information** | | |
| Title | 1 | **PROCalcitonin-based algorithm for antibiotic use in Acute Pancreatitis (PROCAP): The protocol of a randomised controlled trial.**  **[Page 1]** |
| Trial registration | 2a | **ISRCTN 50584992.**  **[Page 1]** |
|  |  |  |
| Protocol version | 3 | **1.0 14^th^ March 2018.**  **[Page 1]** |
| Funding | 4 | **This study has no funding.**  **[Page 21]** |
| Roles and responsibilities | 5a | Names, affiliations, and roles of protocol contributors  **Names:**  Ajith K Siriwardena^1,2^, Santhalingam Jegatheeswaran^1^, James M Mason^3^, Minas Baltatzis^1^, Anthony Chan^1^, Aali J Sheen^1,,4^, Derek O’Reilly^1,2^, Saurabh Jamdar^1^, Rahul Deshpande^1^, Nicola de Liguori Carino^1^, Thomas Satyadas^1^, Ahmed Qamruddin^5^, Katharine Hayden^6^, Michael J Parker^7^, John Butler^7^, Azita Rajai^2^ and Ben McIntyre^8^.  **Affiliations:**  ^1^Regional Hepato-Pancreato-Biliary Unit, ^5^Dept of Microbiology, ^6^Dept of Clinical Biochemistry, ^7^Critical Care Unit and ^8^Pharmacy Department, Manchester Royal Infirmary.  ^2^Faculty of Biology, Health and Life Sciences, University of Manchester & University of Manchester , Manchester Academic Health Sciences Centre.  ^3^Warwick Medical School and University of Warwick, ^4^Centre for Biomedicine, Manchester Metropolitan University.  **Roles of protocol contributors:**  **Ajith K Siriwardena**: Chief Investigator: Conceived the study concept, designed the trial protocol, submitted the IRAS, site specific and ISRCTN applications, designed the case report form, screening log and electronic case report forms; supervisor for data entry, will be involved in final analysis and manuscript writing.  **Santhalingam Jegatheeswaran:** co-designed the trial protocol, helped to submit the IRAS, site specific and ISRCTN applications, co-designed the case report form, co- designed screening log and electronic case report form; recruits patients to study and is responsible for data entry into case report forms.  **James M Mason co-**designed the trial protocol, designed the case report form, designed screening log and electronic case report form; will undertake the final data analysis and acts as Health Economist for PROCAP; writing of final manuscript.  **Minas Baltatzis** co-designed the trial protocol and led literature searches, co-designed the case report form, co-designed screening log and electronic case report form; recruits patients to study and is responsible for data entry into case report forms. **Anthony KC Chan** co-designed the trial protocol, co-designed the case report form, co-designed screening log and electronic case report form; recruits patients to study and is responsible for data entry into case report forms. **Aali J Sheen** Contributed to protocol design, contributed to patient recruitment and clinical management of patients in PROCAP, involved in final manuscript writing. **Derek A O’Reilly** Contributed to protocol design and provided specialist knowledge from NCEPOD review, contributed to patient recruitment and clinical management of patients in PROCAP, involved in final manuscript writing. **Saurabh Jamdar** Contributed to protocol design, reviewed final protocol prior to IRAS submission, contributed to patient recruitment and clinical management of patients in PROCAP, involved in final manuscript writing. **Rahul Deshpande:** Contributed to protocol design, contributed to patient recruitment and clinical management of patients in PROCAP, involved in final manuscript writing. **Nicola de Liguori Carino** Contributed to protocol design, contributed to patient recruitment and clinical management of patients in PROCAP, involved in final manuscript writing. **Thomas Satyadas** Contributed to protocol design, contributed to patient recruitment and clinical management of patients in PROCAP, involved in final manuscript writing. **Ahmed Qamruddin** Contributed to protocol design and provided specialist microbiology input at design stage, involved in definition of endpoints and ensuring that the PCT algorithm was clinically feasible and practical; involved in final manuscript writing. **Katharine Hayden** Contributed to protocol design and provided specialist biochemistry input at design stage, involved in definition of endpoints and ensuring that the PCT algorithm was clinically feasible and practical; involved in final manuscript writing. **Michael J Parker** Contributed expertise to protocol design including advice on the recruitment and management of patients with severe acute pancreatitis in intensive care unit, will be involved in management of patients with severe acute pancreatitis, involved in final manuscript writing. **John Butler:** Conceived study concept, provided expertise from HTA panel perspective on PCT algorithm, involved in protocol design, care of patients with acute pancreatitis in intensive care and final manuscript writing. **Azita Rajai:** Trust statistician: reviewed protocol prior to IRAS submission, helped to define appropriate endpoints and methods of assessment of endpoints; helped to define appropriate sample size; will be involved in final manuscript writing. **Ben McIntyre**: Involved in writing of protocol with special expertise in ensuring that the PCT algorithms and interventions were clinically practical, compliant with Trust protocols and feasible. Involved in care of patients with acute pancreatitis in PROCAP and will be involved in final manuscript writing.  [Page 1] |
|  | 5b | Name and contact information for the trial sponsor  Lynn Webster  Head of Research Office  Manchester University NHS Foundation Trust  Oxford Road  Manchester M13 9RN.  Email: [lynn.webster@mft.nhs.uk](mailto:lynn.webster@mft.nhs.uk)  [Page 21] |
|  | 5c | Role of study sponsor and funders, if any, in study design; collection, management, analysis, and interpretation of data; writing of the report; and the decision to submit the report for publication, including whether they will have ultimate authority over any of these activities  The study sponsor had no involvement in study design. The sponsor is not involved in collection, management, analysis or interpretation of the data.  The sponsor will not be involved in writing the report of the decision to submit the report for publication.  The ultimate authority and responsibility for these activities rests with the Chief Investigator.  [Page 21]. |
|  | 5d | Composition, roles, and responsibilities of the coordinating centre, steering committee, endpoint adjudication committee, data management team, and other individuals or groups overseeing the trial, if applicable (see Item 21a for data monitoring committee)  There is no co-ordinating centre.  PROCAP has a Data Monitoring Committee and a Trial Steering Committee. The composition and role of the DMC is listed in item 21. PROCAP has no endpoint adjudication committee. The data management team are composed of the Chief Investigator and the Clinical Trial Research Fellow.  Trial Steering Committee:  The Trial Steering Committee will have an independent chairperson and an independent member together with the principal investigator, trial co-ordinator and statistician. The TSC will meet 6 monthly.  The DMC will receive and review the progress and accruing data of the trial, providing advice on the conduct of the trial to the Trial Steering Committee. Following each periodic assessment the DMC should inform the Chair of the TSC, in their view, of the ongoing viability of the trial. Each interim review will include updated figures on recruitment, data quality, and safety data. There is no planned interim analysis and thus no interim review of study outcomes.  [Pages 18 and 19]. |
|  |  |  |
| **Introduction**  Background and rationale | 6a | Description of research question and justification for undertaking the trial, including summary of relevant studies (published and unpublished) examining benefits and harms for each intervention  Acute pancreatitis is an inflammatory disorder of the pancreas with an incidence of 150 to 420 cases per million and an overall case-fatality rate of 4-6%. In addition to being a significant cause of death, severe acute pancreatitis (SAP) is associated with prolonged critical care occupancy, lengthy in-patient stay and slow rehabilitation. SAP is characterized by necrosis of pancreatic tissue which with bacterial colonization leads to infected necrosis. Antimicrobial therapy to prevent infection of necrosis in acute pancreatitis has been evaluated in a series of randomized controlled trials with overall findings demonstrating lack of benefit reported in meta-analyses and a Cochrane systematic review. Discriminating between pancreatic infection and inflammation is difficult with neither clinical assessment nor markers of inflammation (such as leukocyte count or C-reactive protein) being sufficiently accurate. As a result there is over-use of antibiotics for suspected infection in acute pancreatitis with up to two-thirds of patients receiving at least one course of antibiotics during their admission. Measurement of procalcitonin can distinguish between bacterial infection and systemic inflammation. The procalcitonin level in the bloodstream of healthy individuals is below the limit of detection (10 pg/mL) using clinical assays. Procalcitonin levels rise rapidly in response to a pro-inflammatory stimulus of bacterial origin and normally fall after successful treatment. PCT is more sensitive than clinical assessment and routine laboratory markers of sepsis (such as leukocyte count and C-reactive protein) in detecting pancreatic infection. Qu and colleagues reported the results of the only randomized controlled trial of a procalcitonin algorithm in severe acute pancreatitis: a single-centre study of 71 patients from China. They compared a PCT-based algorithm for guidance of antibiotic use to routine care in patients with acute pancreatitis. The duration of antibiotic treatment in the PCT-guided group was significantly shorter (10.9 ± 2.8 vs 16.1 ± 2.5 days, p<0.001) without any adverse effects on outcome. Duration of intensive care treatment, overall hospital stay and cost of care were significantly reduced in the PCT-guided group. However, all patients in the control arm were given antibiotics for up to 14 days which does not reflect current international guideline recommendations. Thus the findings of this study need to be reproduced in a setting where antibiotic use follows contemporary practice before procalcitonin-based algorithms can be recommended to guide antibiotic use in acute pancreatitis  [Pages 6-7] |
|  | 6b | Explanation for choice of comparators  The comparator arm in PROCAP will be patients receiving standard care for acute pancreatitis.  [Page 6] |
| Objectives | 7 | Specific objectives or hypotheses  PROCAP tests the hypothesis that a procalcitonin-based algorithm to guide initiation, continuation and discontinuation of antibiotics will lead to reduced antibiotic use in patients with acute pancreatitis without an adverse effect upon outcome.  [page 7] |
| Trial design | 8 | Description of trial design including type of trial (eg, parallel group, crossover, factorial, single group), allocation ratio, and framework (eg, superiority, equivalence, noninferiority, exploratory)  This is a single-centre, randomised, controlled, single-blind, two-arm phase III pragmatic clinical and cost-effectiveness trial. Patients will be allocated on a 1:1 basis to intervention and control. Patients, but not clinicians, will be blind to their allocation.  [Page 9] |
| Methods: Participants, interventions, and outcomes | | |
| Study setting | 9 | Description of study settings (e.g. community clinic, academic hospital) and list of countries where data will be collected. Reference to where list of study sites can be obtained  The study will take place in the in-patient wards of the Manchester Royal Infirmary, a secondary and tertiary care academic medical centre in the United Kingdom.  [Page 9] |
| Eligibility criteria | 10 | Inclusion and exclusion criteria for participants. If applicable, eligibility criteria for study centres and individuals who will perform the interventions (eg, surgeons, psychotherapists)  Inclusion criteria:  Adult patients presenting with acute pancreatitis admitted or referred to the service. Inclusion criteria include:  1. Patients over the age of 18 years of age.  2. Valid informed consent.  3. The diagnosis of acute pancreatitis requires two of the following three features:  I. abdominal pain consistent with acute pancreatitis (acute onset of a persistent, severe, epigastric pain often radiating to the back).  II. serum lipase activity (or amylase activity) at least three times greater than the upper limit of normal.  III. Characteristic findings of acute pancreatitis on contrast-enhanced computed tomography (CECT), magnetic resonance imaging (MRI) or transabdominal ultrasonography.  Exclusion criteria:  1. Patients under the age of 18 years of age.  2. Comorbidities requiring prolonged antibiotic therapy – such as infective endocarditis.  3. Severely immunocompromised patients – such as those with human immunodeficiency virus and with a CD4 count of less than 200 cells/mm^3^; neutropenic patients (<500 neutrophils/mm^3^).  4. Patients on immunosuppressive therapy.  5. Previous thyroid surgery.  [Pages 9 – 10] |
| Interventions | 11a | Interventions for each group with sufficient detail to allow replication, including how and when they will be administered  Intervention  The intervention is the use of a procalcitonin-based algorithm to guide antibiotic use. Patients will be randomised in a 1:1 ratio to receive algorithm-guided or standard care. The randomisation will be stratified by patient admission route (direct or tertiary referral).  Intervention arm protocol  Baseline PCT will be measured on admission (day 0) and the algorithm followed. For patients admitted to ward-based care, PCT will be routinely re-assayed on day 4 and at day 7 after admission for those patients remaining in hospital to these time points. Venesection for PCT assay will be undertaken at the same time as venesection for routine clinical blood tests: no additional venesection is required for PCT measurement. For patients admitted to the critical care unit, PCT will be measured daily during the acute phase of their illness.  Patients who become symptomatic (at any point) for infection will undergo PCT assay and follow the PCT algorithm. Clinically symptomatic patients with a low PCT will not receive antibiotics. If there is persisting concern of infection in patients with a low PCT, the test will be repeated at 24h. Symptomatic patients with a raised PCT will receive antibiotics according to Manchester University Foundation Trust antibiotic policy. The PCT algorithm will be used to guide continuance and discontinuation of antibiotics. In asymptomatic or symptomatic patients with positive microbiology results it is appropriate to treat positive microbiology results with antibiotics. PCT should be measured before commencing antibiotics. PCT measurement should be used to guide cessation of therapy, either after 48h, 72h or 96h as clinically appropriate. To avoid repeated short courses of antibiotics, if antibiotic use is triggered by the algorithm, use will be continued for at least 48h and then PCT re-assayed. If there is no clinical evidence of infection at this point with this second PCT measurement below threshold, antibiotic use will be discontinued. If antibiotics have been prescribed outwith the algorithm then continued use will be discussed with the consultant hepato-pancreato-biliary (HPB) surgeon under whose care the patient is being treated or with the Chief Investigator. After this discussion, antibiotic therapy may be stopped. The Clinician over-ride can be used to either start or stop antibiotics in situations of clinical urgency. Then, the clinician must be either a consultant HPB surgeon or a consultant Intensive Care Physician, and reason for over-ride will be documented. If patients undergo endoscopic, radiological or surgical procedures which would normally be undertaken under the cover of antibiotic prophylaxis it is appropriate to do this without PCT measurement.  [Page 10-11] |
|  | 11b | Criteria for discontinuing or modifying allocated interventions for a given trial participant (eg, drug dose change in response to harms, participant request, or improving/worsening disease).  There is an option to over-ride the protocol and prescribe antibiotics in situations where the procalcitonin level does not recommend either starting or continuing antibiotic treatment. These protocol over-rides must be made in consultation with a consultant-level clinician, either from intensive care or a hepato-pancreato-biliary surgeon.  In addition, patients have the option to withdraw from the study at any point and without providing a reason for doing so. Data collected until that point will be retained and utilised in analysis.  [Page 11] |
|  | 11c | Strategies to improve adherence to intervention protocols, and any procedures for monitoring adherence (eg, drug tablet return, laboratory tests)  Patients in the intervention arm of the trial are clearly identified by a trial sticker in the case notes and drug kardex. Per protocol procalcitonin measurements are pre-ordered in the Trust’s computerised biochemistry test requesting system.  All staff involved in the acute care of in-patients with acute pancreatitis were invited to a pre-launch meeting.  [Page 10] |
|  | 11d | Relevant concomitant care and interventions that are permitted or prohibited during the trial  All standard care for patients with pancreatitis is permitted and is in compliance with the recommendations of the International Association of Pancreatology. The sole exception is that for patients in the intervention arm, all decisions around antibiotic therapy are based on procalcitonin measurement.  [Page 12] |
| Outcomes | 12 | Primary, secondary, and other outcomes, including the specific measurement variable (eg, systolic blood pressure), analysis metric (eg, change from baseline, final value, time to event), method of aggregation (eg, median, proportion), and time point for each outcome. Explanation of the clinical relevance of chosen efficacy and harm outcomes is strongly recommended  i) Primary outcome measure  The primary outcome measure will be the binary outcome: whether antibiotic use occurs during the index stay.  ii) Secondary outcome measures:  1. Safety non-inferiority endpoint all-cause mortality.  2. Days of antibiotic use defined as any day (24 hour period) when antibiotics were prescribed on the patient’s drug prescription chart and administered  3. Clinical infections as defined according to the Centers for Disease Control.  4. New isolates of multi-resistant bacteria (Clostridium difficile, vancomycin resistant enterococcus [VRE], methicillin resistant staphylococcus aureus [MRSA], carbapenemase producing enterobacteriaceae [CPE]).  5. Incidence of multi-resistant organism bacteraemia.  6. Infection of pancreatic necrosis – defined either as a result of fine needle aspiration (FNA), radiological evidence of gas in a peri-pancreatic collection or positive microbiological cultures from surgical or post-mortem specimens.  7. Use of radiological, endoscopic or surgical intervention.  8. Survival at 90 days; time-to-event (mortality) survival (Kaplan-Maier)  9. Length of inpatient stay (in total and by level of care: critical care levels II/III, ward-based care)  10. Re-admission to hospital within 6 weeks of onset of index episode.  11. Episode-related mortality and cause.  12. Quality of life assessed by the EQ-5D-5L questionnaire, at 3 days, discharge and 90 days.  13. Cost analysis (from an NHS perspective, including inpatient resource use).  [pages 12 and 13]/ |
| Participant timeline | 13 | Time schedule of enrolment, interventions (including any run-ins and washouts), assessments, and visits for participants. A schematic diagram is highly recommended (see Figure)  Patients with acute pancreatitis are identified to the research team by their clinical team. They are then approached by the trial research fellow and are provided with information in relation to the trial and have as much time as is required to read this documentation. Once enrolled, they are allocated to either the INTERVENTION or CONTROL arms, For patients in the INTERVENTION arm, all decisions around the use of antibiotics are based on procalcitonin measurement.  A schematic diagram is provided.  [Page 33] |
| Sample size | 14 | Estimated number of participants needed to achieve study objectives and how it was determined, including clinical and statistical assumptions supporting any sample size calculations.  Based on current audit data, 60% of patients admitted with acute pancreatitis receive antibiotics. A 20% absolute change in antibiotic use would be a clinically important difference. This effect of intervention has been observed in other studies evaluating a procalcitonin algorithm to guide antibiotic use. A study with 80% power and 5% significance (2-sided) would require 97 patients in each arm (194 patients in total). The study will aim to recruit 200 patients. Assuming a 3.6% mortality rate based on unit audit data, the sample size provides a 6.6% non-inferiority margin for the safety measure of overall mortality, assuming no change in mortality, 80% power and 95%CI (one-sided).  [Page 14] |
| Recruitment | 15 | Strategies for achieving adequate participant enrolment to reach target sample size  All acute admissions are screened for potential trial participants. This process is undertaken by the trial research fellow and is repeated every 24 hours.  [Page 9] |
| **Methods: Assignment of interventions (for controlled trials)** | | |
| Allocation: |  |  |
| Sequence generation | 16a | Method of generating the allocation sequence (e.g., computer-generated random numbers), and list of any factors for stratification. To reduce predictability of a random sequence, details of any planned restriction (eg, blocking) should be provided in a separate document that is unavailable to those who enrol participants or assign interventions  The allocation sequence is computer generated random numbers using random block sizes of 4, 6 or 8.  Patients are stratified by disease severity (mild or moderate/severe acute pancreatitis) and by admission route (Direct admission to hospital or Tertiary transfer).  [Page 16] |
| Allocation concealment mechanism | 16b | Mechanism of implementing the allocation sequence (e.g., central telephone; sequentially numbered, opaque, sealed envelopes), describing any steps to conceal the sequence until interventions are assigned.  Web-based randomisation will be provided by the Clinical Trials Unit of the University of Edinburgh (https://www.ed.ac.uk/usher/edinburgh-clinical-trials).  [Page 16] |
| Implementation | 16c | Who will generate the allocation sequence, who will enrol participants, and who will assign participants to interventions  The allocation sequence is generated by the Clinical Trials Unit of the University of Edinburgh.  The trial clinical research fellow, Mr Santhalingam Jegatheeswaran will enrol participants. Cross-cover is provided by Dr Minas Baltatzis. Mr Jegatheeswaran or Dr Baltatzis will assign patients to either arm according to the allocation sequence.  [Page 16] |
| Blinding (masking) | 17a | Who will be blinded after assignment to interventions (eg, trial participants, care providers, outcome assessors, data analysts), and how.  Patients are blind to the arm to which they are allocated. There is no trial-arm identifiable information in the folders at the foot of the patient’s bed.  Care providers and outcome assessors are not blind to the allocation arm.  [Page 9] |
|  | 17b | If blinded, circumstances under which unblinding is permissible, and procedure for revealing a participant’s allocated intervention during the trial.  It is possible to see which arm the patient is allocated to by review of the case notes. The notes and drug kardex will contain a sticker indicating whether the patient is in the INTERVENTION arm.  {Pages 10,11]. |
| **Methods: Data collection, management, and analysis** | | |
| Data collection methods | 18a | Plans for assessment and collection of outcome, baseline, and other trial data, including any related processes to promote data quality (eg, duplicate measurements, training of assessors) and a description of study instruments (eg, questionnaires, laboratory tests) along with their reliability and validity, if known. Reference to where data collection forms can be found, if not in the protocol  Information on screening and recruitment is recorded in an electronic screening log. This provides information on all patients screened for recruitment and reasons for non-recruitment. Once recruited clinical trial data are recorded in real-time in a case-report form (CRF). After discharge, information from the paper-based CRF is transferred onto an electronic dataset master copy. Quality of life assessment at enrolment, prior to discharge from hospital and at 90 days is undertaken using the EQ-5DL form.  [Page 17] |
|  | 18b | Plans to promote participant retention and complete follow-up, including list of any outcome data to be collected for participants who discontinue or deviate from intervention protocols.  There are no specific plans to promote participant retention. PROCAP is a study which takes place during the acute phase of hospital admission and data entry (apart from the day 90 EQ-5DL) is complete by the time of discharge from hospital.  [no match in protocol]. |
| Data management | 19 | Plans for data entry, coding, security, and storage, including any related processes to promote data quality (eg, double data entry; range checks for data values). Reference to where details of data management procedures can be found, if not in the protocol  Information on screening and recruitment is recorded in an electronic screening log. Once recruited clinical trial data are recorded in real-time in a case-report form (CRF). After discharge, information from the paper-based is transferred onto an electronic dataset master copy. The electronic screening log and dataset master copy are stored on a password-protected NHS computer sited in a locked office. The paper forms are stored in the same office.  [Page 17] |
| Statistical methods | 20a | Statistical methods for analysing primary and secondary outcomes. Reference to where other details of the statistical analysis plan can be found, if not in the protocol  Clinical and economic analysis will follow intention-to-treat principles, as detailed prospectively. Endpoints will be assessed using appropriate general linear model adjusted for stratification factors; for the primary endpoint a general linear regression with logit link will be employed. Missing values with be addressed by multiple imputation, having appropriately explored the missingness mechanism, and in accordance with good practice. Chance baseline imbalances and protocol adherence will be explored within sensitivity analyses.  [Page 17] |
|  | 20b | Methods for any additional analyses (eg, subgroup and adjusted analyses)  There are no planned additional subgroup analyses. |
|  | 20c | Definition of analysis population relating to protocol non-adherence (eg, as randomised analysis), and any statistical methods to handle missing data (eg, multiple imputation)  See 20a |
| **Methods: Monitoring** | | |
| Data monitoring | 21a | Composition of data monitoring committee (DMC); summary of its role and reporting structure; statement of whether it is independent from the sponsor and competing interests; and reference to where further details about its charter can be found, if not in the protocol. Alternatively, an explanation of why a DMC is not needed  **Data Monitoring Committee**  Members:  Chair – Giuseppe Garcea (Leicester).  Philip Martin – Patient advocate.  Catherine Fullwood (Statistician).  The CI may be asked, and should be available, to attend open sessions of the DMC meeting. The other TSC members will not usually be expected to attend but can attend open sessions when necessary.  **Roles and responsibilities:**  The aim of the committee is to safeguard the interests of trial participants, assess the safety and viability of the intervention during the trial, and monitor the overall conduct of the clinical trial. The DMC will receive and review the progress and accruing data of the trial, providing advice on the conduct of the trial to the Trial Steering Committee [TSC].Following each 6 monthly assessment the DMC should inform the Chair of the TSC, in their view, of the ongoing viability of the trial. Each interim review will include updated figures on recruitment, data quality, and safety data. There is no planned interim analysis and thus no interim review of study outcomes. Specific aspects include:  •assess data quality, including completeness.  •monitor recruitment figures  •monitor compliance with the protocol by participants and investigators  •monitor evidence for treatment harm (e.g., SAEs, deaths, deep infections & infection with resistant bacteria)  •decide whether to recommend that the trial continues to recruit participants or whether recruitment should be terminated either for everyone or for some participant subgroups  •monitor compliance with previous DMC recommendations  •consider the ethical implications of any recommendations made by the DMC  •suggest additional data analyses  •advise on protocol modifications suggested by investigators  •monitor appropriateness of patient information  •assess the impact and relevance of external evidence.  [Pages 18 and 19]. |
|  | 21b | Description of any interim analyses and stopping guidelines, including who will have access to these interim results and make the final decision to terminate the trial.  No interim analyses are scheduled.  Stopping guidelines:  The trial may be stopped temporarily or permanently (following discussion with the DMC and sponsor) at any point if :  1. There is evidence of trial misconduct noted by the DMC or by the MFT Research and Innovation Department.  2. If there is evidence of futility or if there is evidence that the safety non-inferiority endpoint is not met.  3. If external scientific evidence emerges to render the findings of this trial obsolete or irrelevant.  [Pages 19 and 20] |
| Harms | 22 | Plans for collecting, assessing, reporting, and managing solicited and spontaneously reported adverse events and other unintended effects of trial interventions or trial conduct  All adverse events will be assessed for  1. Seriousness  2. Causality  3. Expectedness  The research fellow will notify the principle investigator of the adverse event. The principle investigator will determine whether it is an adverse event (AE), serious adverse event (SAE) or serious adverse reaction (SAR). All adverse events will be recorded in line with European Directive 2001/20/EC and recorded in the case report form. An annual safety report to the data monitoring committee will be submitted. Serious adverse events (SAEs) will be reported by email to the Trust. SAEs will be reported quarterly.  [Page 19] |
| Auditing | 23 | Frequency and procedures for auditing trial conduct, if any, and whether the process will be independent from investigators and the sponsor  The Data Monitoring Committee will meet on a 6 monthly basis. The DMC is independent of the investigators and sponsor. The DMC will have access to trial data and will report to the TSC.  [pages 18 and 19] |
| Ethics and dissemination | | |
| Research ethics approval | 24 | Plans for seeking research ethics committee/institutional review board (REC/IRB) approval  The study was approved by the NHS Health Research Authority (REC reference 18/NW/0255) on the 29th May 2018.  Site specific approval was granted by the Manchester University Foundation Trust (Pin B00007) on the 5th June 2018.  PROCAP was registered with the International Standard Randomised Controlled Trial Number (ISRCTN 50584992) on the 7th February 2018 prior to opening the study for recruitment.  The study opened for recruitment on the 26^th^ July 2018 and is intended to recruit for 24 months.  [Page 20] |
| Protocol amendments | 25 | Plans for communicating important protocol modifications (eg, changes to eligibility criteria, outcomes, analyses) to relevant parties (eg, investigators, REC/IRBs, trial participants, trial registries, journals, regulators)  Any protocol modifications will be recorded by modification of the study protocol number and will be submitted to the North West Research Ethics Committee as an amendment. |
| Consent or assent | 26a | Who will obtain informed consent or assent from potential trial participants or authorised surrogates, and how (see Item 32)  Consent process.  Valid consent will be obtained for all patients. Consent procedures will be governed by the Medicines for Human Use (Clinical Trials) Regulations (2004); Schedule 1, part 5 and by The Medicines for Human Use (Clinical Trials) Amendment (No.2) Regulations 2006, No. 2984.[33,34] Where available, Trust-appointed translator services will be used for those patients who are unable to speak or comprehend English.  Informed consent for patients with capacity  For eligible patients who possess mental capacity, a member of the research team will make the initial approach and provide a verbal overview of the study and what participation will involve. The patient will be provided with a written information sheet and given the opportunity to ask questions. After their questions have been answered, they have had sufficient time to consider participation and if they are willing to take part in the study, they will be asked to sign the consent form.  Consent procedures for patients who lack capacity  As acute pancreatitis maybe severe, causing disruption of a patient’s cognitive state or requiring sedation to facilitate advanced organ support in intensive care, some potential participants will lack capacity to consent for enrolment. Such patients may still be enrolled in this study according to the following procedures. Firstly, a treating clinician who is not part of the study team will assess the competence of a potential participant to consent for research. If lack of capacity to consent is confirmed then valid consent for enrolment may be obtained from a patient’s legal representative. Ideally this legal representative will be someone who knows the patient and is able to judge whether the patient would have agreed to enrolment in this study. This personal legal representative would usually be their next of kin or someone with whom they had a significant relationship, and is willing to engage with the consent process on the patient’s behalf. If a personal legal representative is not available then the patient’s professional legal representative may provide consent instead: an independent treating clinician who is not part of the study team. Where a researcher is also the treating health professional, another member of the research team, independent of any responsibility for the clinical care of that patient, will be asked to make the initial approach and/or seek consent from participants or their legal representative. Patients who recover sufficiently to understand the explanation of the study will be asked to consent to continue in the study procedures as soon as possible or be offered the chance to withdraw. If the patient chooses to withdraw from the study procedures, they will be asked for permission to use their study-related data and for permission to collect and use outcome data.  [Pages 14-16] |
|  | 26b | Additional consent provisions for collection and use of participant data and biological specimens in ancillary studies, if applicable  There are no plans for additional use of participant data. No biological specimens will be used for research purposes. |
| Confidentiality | 27 | How personal information about potential and enrolled participants will be collected, shared, and maintained in order to protect confidentiality before, during, and after the trial  All data are anonymised and patients are identifiable only by trial number. Data are stored in an encrypted data base on a password protected computer in a locked NHS office. Data will be discarded 12 months after completion of this trial.  [Page 17] |
| Declaration of interests | 28 | Financial and other competing interests for principal investigators for the overall trial and each study site  There are no financial or any other competing interests for any of the co-authors. |
| Access to data | 29 | Statement of who will have access to the final trial dataset, and disclosure of contractual agreements that limit such access for investigators.  The final trial dataset will be available to the Chief Investigator and Professor James Mason.  There are no contractual agreements to permit access to these data to third parties. |
| Ancillary and post-trial care | 30 | Provisions, if any, for ancillary and post-trial care, and for compensation to those who suffer harm from trial participation.  Crown indemnity will apply to participants. |
| Dissemination policy | 31a | Plans for investigators and sponsor to communicate trial results to participants, healthcare professionals, the public, and other relevant groups (eg, via publication, reporting in results databases, or other data sharing arrangements), including any publication restrictions  The results will be presented at appropriate national and international scientific meetings and published in a peer-reviewed journal. |
|  | 31b | Authorship eligibility guidelines and any intended use of professional writers  ICJME criteria for authorship will be applied. There is no intention to use professional writers. |
|  | 31c | Plans, if any, for granting public access to the full protocol, participant-level dataset, and statistical code  The protocol will be published. The full dataset and statistical code will be made available as an online supplement to the final published report. |
| Appendices |  |  |
| Informed consent materials | 32 | Model consent form and other related documentation given to participants and authorised surrogates  The following study consent related documents are provided in the appendices:  Appendix 1: Participant Information Leaflet.  Appendix 2: Participant Consent form.  Appendix 3: Letter to Participant’s General Practitioner.  Appendix 4: Consultee information leaflet.  Appendix 5: Consultee declaration. |
| Biological specimens | 33 | Plans for collection, laboratory evaluation, and storage of biological specimens for genetic or molecular analysis in the current trial and for future use in ancillary studies, if applicable  Not applicable to PROCAP. |

*It is strongly recommended that this checklist be read in conjunction with the SPIRIT 2013 Explanation & Elaboration for important clarification on the items. Amendments to the protocol should be tracked and dated. The SPIRIT checklist is copyrighted by the SPIRIT Group under the Creative Commons “[Attribution-Non-commercial-OneDrives 3.0 Unported](http://www.creativecommons.org/licenses/by-nc-nd/3.0/)” license.
