# Supplementary material for: PROCalcitonin-based algorithm for antibiotic use in Acute Pancreatitis (PROCAP): study protocol for a randomised controlled trial
Source: Trials. 2019 Jul 29;20:463. doi: 10.1186/s13063-019-3549-3 (PMC6664733; doi:10.1186/s13063-019-3549-3)
Supplement: Supplementary file 3 — Information to GP (DOCX 22 kb) [file 13063_2019_3549_MOESM3_ESM.docx]

# INFORMATION TO GP

**<Headed paper>**

Department of HPB Surgery
Manchester Royal Infirmary

**Affix Patient Sticker**

Oxford Road

Manchester M13 9WL

**Tel:** (0161) 276 4244

**Fax:** (0161) 276 4530

**Date:** /	/2000

Dear Dr ………………………

The above patient has been admitted to Manchester Royal Infirmary with acute pancreatitis. They have given their consent to be enrolled into the following research study currently being carried out by the unit.

**Title:	PROCalcitonin-based algorithm for antibiotic use in Acute Pancreatitis (PROCAP): A randomised controlled trial**

**Principal Investigator:**	**Professor Ajith K Siriwardena**

**Ethics Approval Ref:	XXXXXXXX**

PROCAP is a randomised controlled trial evaluating an algorithm based on serum Procalcitonin levels to guide antibiotic use in patients with acute pancreatitis. In this study, all patients will have standard care with the sole exception being that those randomly allocated to the intervention arm will have antibiotic treatment guided by serum procalcitonin levels. The patients’ involvement in this study ends after they are discharged.

A full clinical discharge summary (separate from this letter) will be sent to you in due course. Please do not hesitate to contact a member of the HPB research team if you have any queries about this research study.

Yours sincerely

Clinical researcher *supervised by Professor A K Siriwardena.*
